# Supplementary material for: Ocular Inflammation Induced by Immune Checkpoint Inhibitors
Source: J Clin Med. 2022 Aug 25;11(17):4993. doi: 10.3390/jcm11174993 (PMC9456546; doi:10.3390/jcm11174993)
Supplement: Supplementary file 1 [file jcm-11-04993-s001.zip › jcm-1881715-supplementary.pdf]

Journal of
Clinical Medicine

Article

Ocular Inflammation Induced by Immune Checkpoint Inhibitors

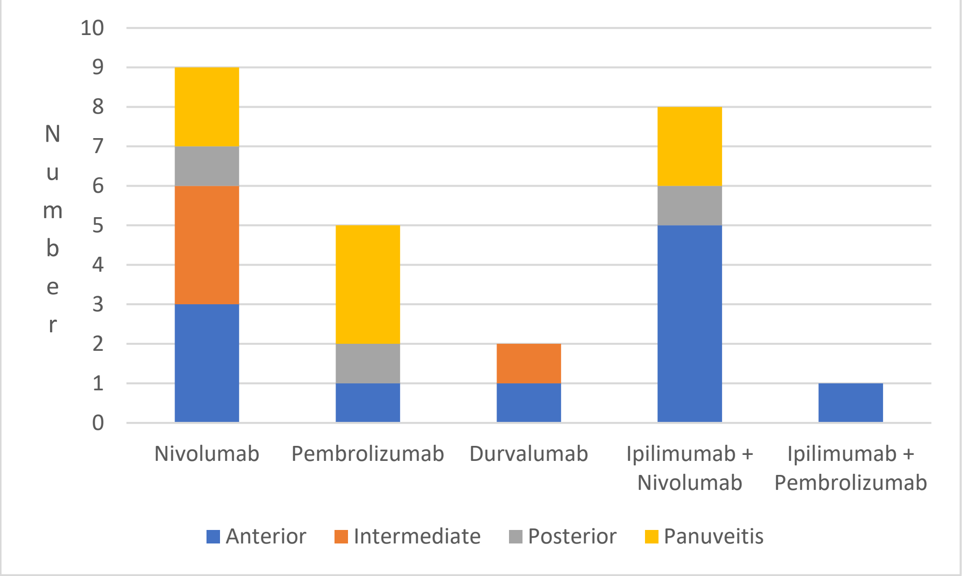

Figure S1. Distribution of uveitis depending on the ICPI

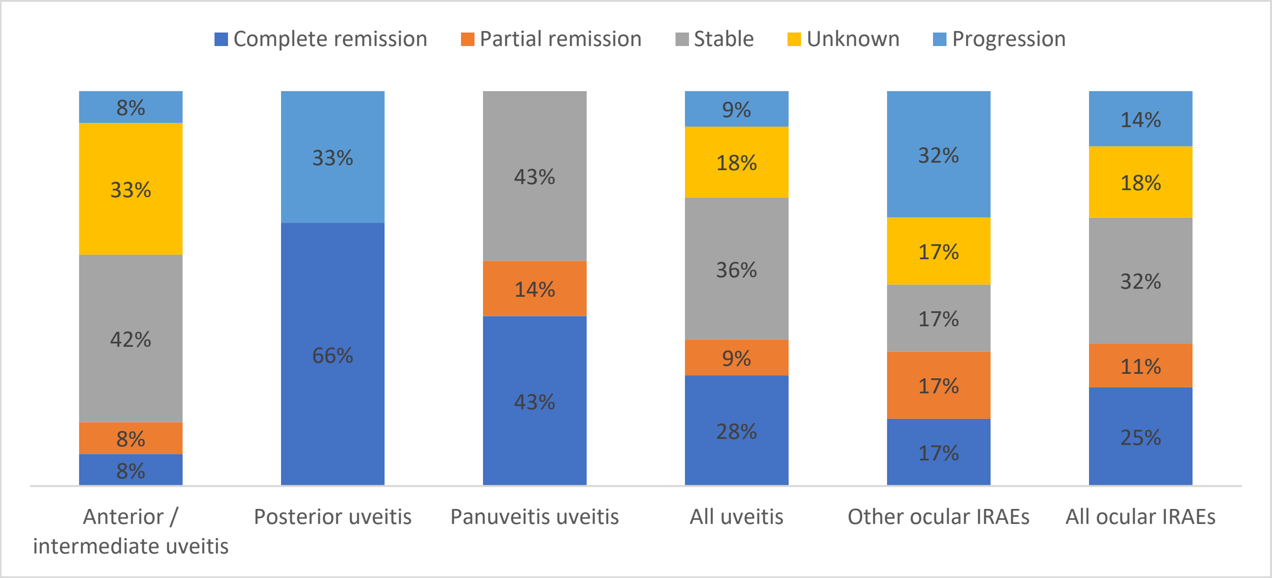

Figure S2. Neoplastic outcome depending on the type of ocular IRAEs at the end of the follow-up.

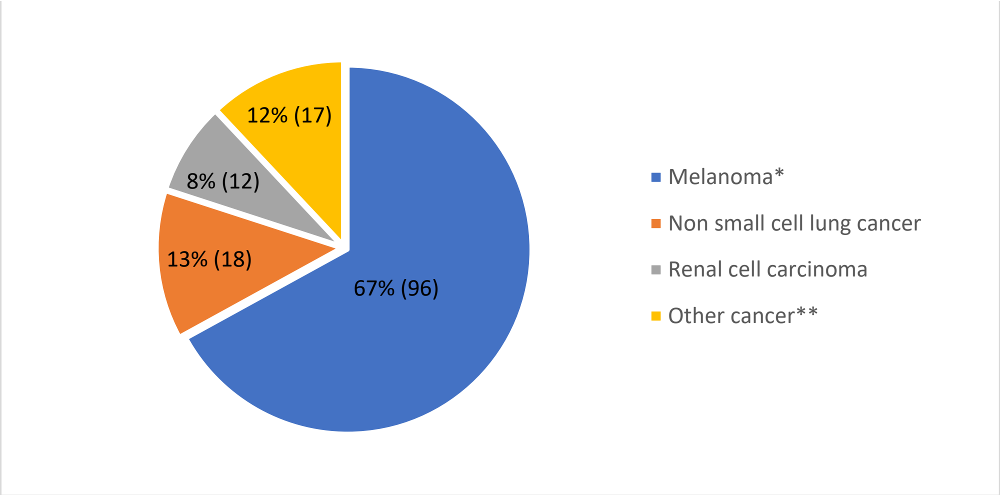

Figure S3. ICPI indications
* 96 patients with melanoma (of whom 7 with uveal melanoma) ;
** Small cell lung cancer (n=2 patients), lymphoma (n=2), prostate carcinoma (n=2), eosophageal carcinoma (n=2), ovarian carcinoma (n=2), endometrial cancer (n=1), colon adenocarcinoma (n=1), hypopharyngeal carcinoma (n=1), hypopharyngeal carcinoma (n=1), duct carcinoma (n=1), cutaneous squamous cell carcinoma (n=1), mesothelioma (n=1), unknown primary neoplasm (n=1).

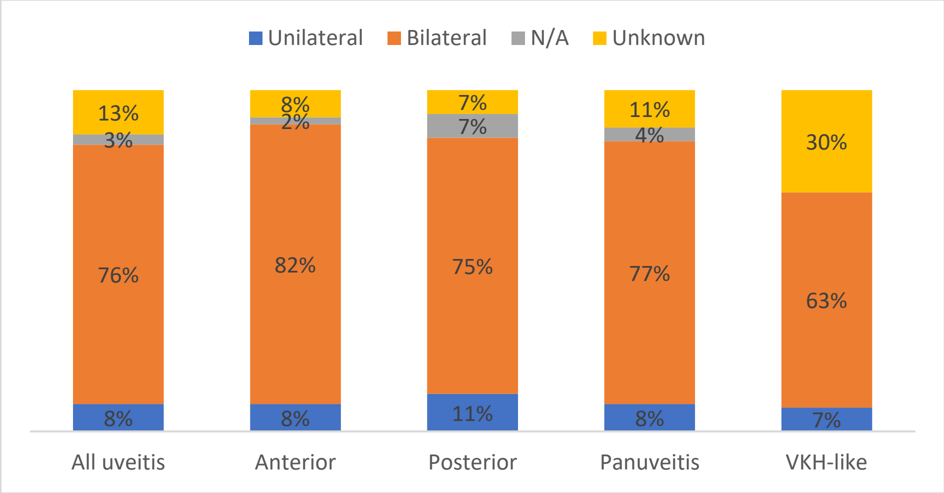

Figure S4. Unilateral or bilateral nature of the uveitis, depending on the type of uveitis.
N/A= not applicable, patients with a history of previous enucleation.

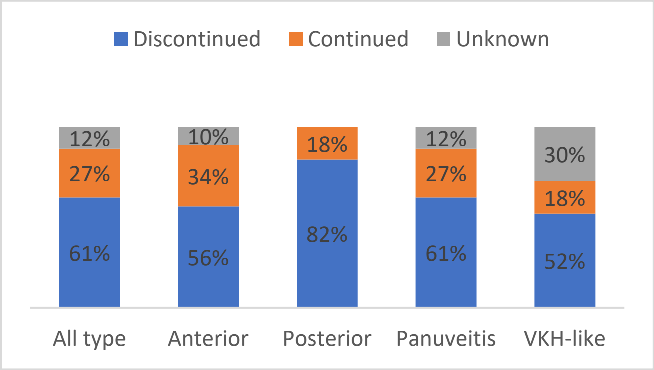

(a)

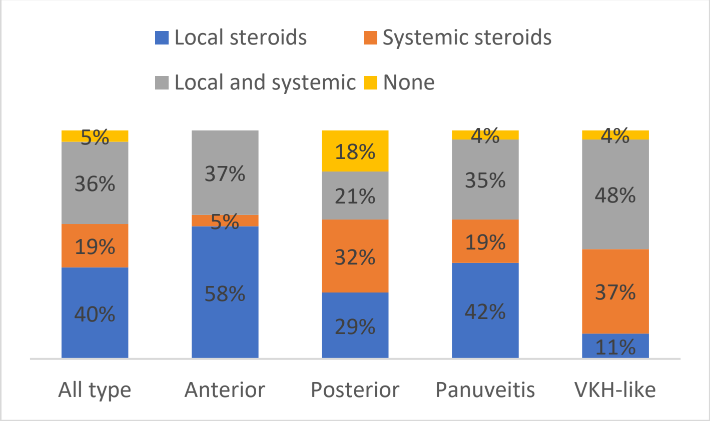

(b)

Figure S5. Discontinuation of ICPI (a) and treatment (b) depending on the type of uveitis.

Table S1. Repartition of uveitis depending on ICPI.

ICPI	All type, n (%)	Anterior, n (%)	Posterior, n (%)	Panuveitis, n (%)	VKH-like, n (%)
Atezolizumab	4 (2.7)	1 (1.6)	2 (7.1)	-	1 (3.7)
Cemiplimab	1 (0.6)	-	1 (3.5)	-	-
Durvalumab	3 (2.0)	2 (3.2)	1 (3.5)	-	-
Ipilimumab	28 (19.5)	16 (25.8)	5 (17.8)	2 (7.6)	5 (18.5)
Ipilimumab + Nivolumab	26 (18.8)	15 (24.1)	3 (10.7)	5 (19.2)	3 (11)
Ipilimumab + Pembrolizumab	2 (1.3)	1 (1.6)	1 (3.5)	-	-
Nivolumab	41 (28.6)	19 (30.6)	6 (21.4)	6 (23)	10 (37)
Pembrolizumab	38 (26.5)	8 (12.9)	9 (32.1)	13 (50)	8 (29.6))
